# Defining the optimal dose of radiation in leukemic patients with extramedullary lesions

**DOI:** 10.1186/1471-2407-11-428

**Published:** 2011-10-06

**Authors:** Jin Ho Song, Seok Hyun Son, Ju Hwan Lee, Su Mi Chung, Hong Seok Jang, Byung Ock Choi

**Affiliations:** 1 Department of Radiation Oncology, College of Medicine, The Catholic University of Korea, Seoul, Korea

## Abstract

**Background:**

Analysis of the clinical response of extramedullary lesions in leukemic patients treated with radiation therapy (RT) and defining the optimal dose of radiation.

**Methods:**

Forty-two extramedullary lesions found in 24 leukemic patients treated with RT were reviewed. The radiation was delivered usually 2 Gy/day, up to a median of 20 Gy (range: 18.0-40.8). The clinical response and symptom palliation effect were analyzed. The factors affecting the response were also included in the analysis.

**Results:**

After a median time of 7.9 weeks, the overall response rate was 76.2%. A complete response (CR) was achieved in 35.7%, a partial response in 40.5%. The symptom was relieved in 85.7% sites. The overall response rate was better in patients whose initial tumor size was smaller than 10 cm^2 ^(*p = 0.010*) or who were treated with more than 25 Gy (*p = 0.031*). The overall CR rate was also higher in those who had smaller tumors (smaller than 6 cm or 30 cm^2^) (*p = 0.015)*, or when the tumor was located in soft tissue (*p = 0.029*).

**Conclusions:**

Extramedullary lesions in leukemic patients can be successfully treated with RT. The tumor response rate was excellent and symptom relief was achieved in almost all patients. There was a better response to treatment when the tumor was small or it was located in soft tissue. Although, there was no definite correlation between volume reduction and total dose, it seems that higher total dose more of than 25 Gy is needed for better response.

## Background

Cure of leukemia is possible for significant numbers of patients after intensive treatment. However, clinical extramedullary lesions (EML) in leukemic patient negatively affect the prognosis [1]. EML in leukemic patients can occur at any age and in any organ or tissue throughout the body, especially soft tissue, skin, bones and lymph nodes [1-4]. However, it can also occur at other sites such as the gastrointestinal tract, genitourinary tract, heart, orbit and the sanctuary area, such as the testis and central nervous system [5-12].

The relatively rare incidence of EML has resulted in limited treatment experience, and there are no publications on randomized trials. Therefore, therapeutic decisions are usually based on retrospective studies and case reports. Chemotherapy is the most important treatment method [1,13,14]. However, on an individual basis, other modalities including surgical decompression or radiation therapy (RT) are sometimes clinically necessary [2,3,14-16].

RT is effective and can be used for localized lesions that cause symptoms [13-17]. However, there is no literature specifying guidelines for radiation oncologists in the treatment of patients with EML. As a result, the dose or fractionation schedule and radiation treatment method varies among institutions and patients. Therefore, we reviewed the clinical response of EML treated by RT.

## Methods

### Patients and tumor characteristics

We retrospectively reviewed the database at the St. Mary's Hospital, College of Medicine, the Catholic University of Korea from between January 2003 and December 2008. There were 37 patients and 88 sites treated with RT. The median follow-up time was 12.8 months (range: 1.9-80.2). To obtain objective clinical response results, we analyzed 24 patients and 42 sites whose follow up imaging study exists. There were 11 males and 13 female patients with a median age of 30 years old (range: 5-69). Most of the patients had acute myeloid leukemia (AML) as a primary disease (75.0%) and the others were acute lymphcytic leukemia (ALL) (20.8%) or CML (chronic myeloid leukemia) (4.2%) patients.

In seven (29.2%) patients, they developed before achieving the first complete remission. Of these patients, two patients had EML initially at the time of the leukemia diagnosis. In eleven (45.8%) patients, EML developed as a first sign of the first leukemia relapse. Of the rest of patients, EML developed during or after the treatment for leukemia relapse. There were 14 patients with a history of bone marrow transplantation and 9 patients receiving a total body irradiation for the conditioning.

The diagnostic workup study included history taking, physical examination and imaging studies. Computed tomography (CT) was acquired in 13 sites and magnetic resonance imaging (MRI) was performed for 29 sites. Of the 42 sites, 14 (33.3%) sites were pathologically confirmed to granulocytic sarcoma. In the other sites, EML was diagnosed based on clinical evidences. Nineteen (45.2%) tumors were located in bone, which was the most commonly affected site. Of the remaining 16 (38.1%) tumors were located in soft tissue, 5 (11.9%) in lymph nodes and 2 (4.8%) in solid organs (one in brain, one in testis). The initial median tumor size was 4.8 cm (range: 1.1-15.0) or 10.1 cm^2 ^(range: 0.9-54.0). The initial median tumor volume was 43.3 cm^3 ^(range: 1.5-247.5). The patients and tumor characteristics are summarized in Table 1.

**Table 1:** Patients and tumor characteristics

Patients Characteristics	No (%)
Gender	
	
Male	11 (45.8)
Female	13 (54.2)

Age, median (range)	30 (5-69)

Primary disease	
	
AML	18 (75.0)
ALL	5 (20.8)
CML	1 (4.2)

Disease pattern	
	
Before 1^st ^remission	7 (29.2)
First manifestation of 1^st ^relapse	11 (45.8)
After relapse	6 (25.0)

History of SCT (+)	14 (58.3)

History of TBI (+)	9 (37.5)

Biopsy confirmed (+)	14 (33.3)

Tumor site	
	
Bone	19 (45.2)
Soft tissue	16 (38.1)
Lymph nodes	5 (11.9)
Solid organ	2 (4.8)

Initial tumor size (LD), cm median (range)	4.8 (1.1-15.0)
	
Initial tumor size (BP), cm^2 ^median (range)	10.13 (0.88-54.0)
	
Initial tumor volume, cm^3 ^median (range)	43.3 (1.5-247.5)

Total	24 patients, 42 sites

*Abbreviations: *AML = Acute myeloid leukemia, ALL = Acute lymphocytic leukemia, CML = Chronic myeloid leukemia, SCT = Stem cell transplantation, TBI = Total body irradiation, LD = Largest dimension, BP = Bidimensional product

### Treatment characteristics

Radiation was delivered usually once daily at 2 Gy, up to a median of 20 Gy (range: 18-40.8) with 6 or 10 MV photon beams. The daily dose was lower than 2 Gy (1.7 or 1.8 Gy) for 10 sites, 2 Gy for 31 sites and 2.5 Gy in only one site. The overall median treatment time was 2.1 weeks (range: 0.9-6.0). Concurrent chemotherapy was delivered during the RT in 9 sites. Four patients received AML-like intensive chemotherapy, 3 patients received intra-thecal chemotherapy and 2 patients received imatinib.

### Response evaluation

The size of tumors was measured on initial diagnostic CT or MRI images. The largest tumor dimension (LD), as well as the dimension perpendicular to it, was measured on transaxial CT or MRI images. The bidimensional tumor product (BP) was calculated as a product of the largest dimension and the dimension perpendicular to it. However, there were some limitations, such as some tumors were large in the cranio-caudal axis. To compensate for this problem, the tumor was contoured with a modern radiation treatment planning system (CorePLAN, Seoul C&J Inc., Korea) to calculate the tumor volume.

Post-therapy CT or MRI images were checked in the same way. Although the follow up period was not consistent in all tumors, the tumor response evaluation was performed according to RECIST criteria. The volume reduction rate was also calculated. The clinical or therapeutic factors that could have influenced the response were analyzed.

### Statistical analysis

The treatment response was analyzed using the following factors: primary disease, tumor site, the use of chemotherapy, initial tumor size, follow up time and the total dose. Chi-square or Fischer's exact test were performed to determine if these factors influenced the response. Student's t-test was used for average comparison, and the Kaplan and Meier method was used for survival analysis. Linear logistic regression methods were used to analyze the relationship between the tumor dose and volume reduction rate. All results were considered statistically significant at the level of *p *< 0.05 in a two-tailed test. SPSS ver. 12.0 for windows (SPSS Inc, Chicago, Illinois) was used for statistical analysis.

## Results

### Tumor Response

The first response evaluation was performed at a median of 1.9 weeks (range: 0-29.7) after the RT. There were 11 sites which the response evaluation was done directly after the RT. There were 12 (28.6%) sites with a complete response (CR) and 17 (40.5%) sites with a partial response (PR). The response rate (CR+PR) was 69.1%. The second response evaluation was able to be acquired in only 20 sites. The median time was 11.7 weeks (range: 2.9-28.4) after RT. In comparison to first response evaluation, there were 6 sites that had a better response (PR to CR in 3 sites, SD to PR in 3 sites), and 12 sites showed the same results. However, two sites progressed. The one was a tumor on the anterior rib, which showed a PR the day after the RT (20.4 Gy in 12 fracions), but progressed in the second evaluation which was done 18.9 weeks after the RT. The other was a tumor on the distal femur which showed SD in the first evaluation (6.6 weeks after RT, 19.8 Gy in 11 fractions), but progressed in second evaluation (15.9 weeks after RT).

The overall response evaluation results are shown in Table 2. The median time for overall response evaluation was 7.9 weeks (range: 0-29.7). The overall CR was at 15 (35.7%) sites, PR was at 17 (40.5%) sites and SD at 8 (19.0%) sites.

**Table 2:** Tumor response rate (number of sites (%))

	1^st ^response (42 sites)	2^nd ^response (20 sites)	Overall response (42 sites)
CR	12 (28.6)	(from PR group) +3	15 (35.7)
PR	17 (40.5)	(from SD group) +3	17 (40.5)
SD	13 (30.9)		8 (19.0)
PD	0 (0.0)	(from SD group) +2	2 (4.8)

Time after RT (weeks)*	1.9 (0.0-47.7)	11.7 (2.9-28.4)	7.9 (0.0-47.7)

*Abbreviations: *CR = Complete response; PR = Partial response; SD = Stable disease; PD = Progressive disease

*median (range)

### Symptom palliation

The symptom score was retrospectively evaluated in four scales determined by reading the chart notes. The pain or symptom was scored 0 to 3 from no pain or symptom, to mild, moderate or severe pain or symptom. The pain was relieved at 36 (85.7%) sites and disappeared at 16 (38.1%) sites. A decrease in the pain score of more than 2 points was observed in 17 patients and a decrease in 1 point in 19 patients. There were no significant differences in symptom palliation among the tumor sites (Table 3).

**Table 3:** The change of pain score after RT according to tumor sites (number of sites (%))

Decrease of pain score	Bone	Soft tissue	Lymph nodes	Solid organ
≥ 2 points	6 (31.6)	8 (50.0)	2 (40.0)	1 (50.0)
1 point	10 (52.6)	7 (43.8)	1 (20.0)	1 (50.0)
No Change	3 (15.8)	1 (6.2)	2 (40.0)	0 (0.0)

Disappeared	4 (21.1)	9 (56.3)	2 (40.0)	1 (50.0)

Total	19	16	5	2

### Factors affecting tumor response

There was no specific correlation between the initial tumor size and total radiation dose. The Pearson R^2 ^was 0.046 for LD and total dose and 0.039 for BP and total dose, which suggests that the total dose was not increased according to initial tumor size. However, the tumor response was differed according to the initial tumor size and the total dose. The overall response rate was better in patients whose initial tumor size (BP) was smaller than 10 cm^2 ^or in those patients treated with more than 25 Gy or BED_10 _30 Gy. Tumors received more than 25 Gy showed a 94.1% (16/17) response rate. In contrast, the overall response rate was only 64.0% (16/25) with less than 25 Gy.

The overall CR rate was not affected by the total dose, but it was lower for those who had larger tumors. When the tumor was larger than 6 cm in LD or larger than 30 cm^2 ^in BP, the CR rate was lower. Of the 13 tumors larger than 6 cm only one tumor showed a CR. However, a CR was achieved in half (14/29) of the small tumors less than 6 cm. The tumor location was also important in the overall CR rate. When the tumor was located in soft tissue, a CR was better achieved than at other sites. The factors affecting the tumor response are listed in Table 4 and Table 5. The use of concurrent chemotherapy did not increase the response rate.

**Table 4:** Factors affecting in overall response rate

Factors ( 2 × 2 analysis)		Response (CR + PR)	Non-response (SD + PD)	Total	*p*-value
Primary disease	Yes	23	4	27	0.128
AML	No	9	6	15	

RT site	Yes	14	2	16	0.270
Soft tissue	No	18	8	26	

Concurrent	Yes	8	1	9	1.000
Chemotherapy	No	24	9	33	

Initial size	Yes	13	9	22	0.010*
BP ≥ 10 cm^2^	No	19	1	20	

Initial size	Yes	14	7	21	0.277
LD ≥ 6.0 cm	No	18	3	21	

Follow up time	Yes	23	7	30	1.000
≥ 2 weeks	No	9	3	12	

Total dose	Yes	16	1	17	0.031*
≥ 25 Gy	No	16	9	25	

BED_10_	Yes	17	1	18	0.026*
≥ 30 Gy	No	15	9	24	

*Abbreviations: *AML = Acute myeloid leukemia, BP = Bidimensional product, LD = Largest dimension, BED = Biologic effective dose

*Statistically significant factors

**Table 5:** Factors affecting in overall CR rate

Factors ( 2 × 2 analysis)		CR (15 sites)	Non-CR (27 sites)	Total	*p*-value
Primary disease	Yes	10	17	27	0.810
AML	No	5	10	15	

RT site	Yes	9	7	16	0.029*
Soft tissue	No	6	20	26	

Concurrent	Yes	3	7	10	1.000
Chemotherapy	No	12	20	32	

Initial size	Yes	0	7	7	0.038*
BP ≥ 30 cm^2^	No	15	20	35	

Initial size	Yes	1	12	13	0.015*
LD ≥ 6.0 cm	No	14	15	29	

Follow up time	Yes	13	17	30	0.158
≥ 2 weeks	No	2	10	12	

Total dose	Yes	8	9	17	0.326
≥ 25 Gy	No	7	18	25	

BED_10_	Yes	8	10	18	0.347
≥ 30 Gy	No	7	17	24	

*Abbreviations: *CR = Complete response, ML = Acute myeloid leukemia, BP = Bidimensional product, LD = Largest dimension, BED = Biologic effective dose

*Statistically significant factors

### Volume reduction rate

**Figure 1:**
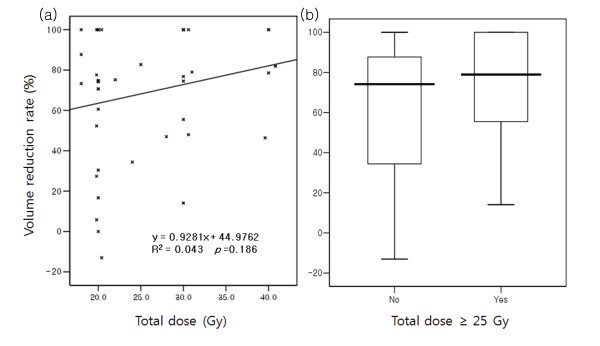
**The volume reduction rate according to the total radiation dose**. (a) Linear logistic regression analysis between the total dose and volume reduction rate. (b) The box-plot of the average volume reduction rate according to total dose.

The initial median volume of the tumor was 43.3 cm^3^. The median volume decreased to 11.4 cm^3 ^and 3.3 cm^3 ^in the first and second response evaluations. The median volume reduction rate was 71.9% and 76.8%, respectively. The overall median volume reduction rate was 74.7%. The volume increased 13.0% and 83.1% in two sites, which were counted as a PD in response evaluation. There were no statistically significant factors affecting the volume reduction rate (Table 6). Although there was no definite linear correlation between the total dose and volume reduction rate, the average volume reduction rate was higher in those who received more than 25 Gy (Figure 1).

**Table 6:** Factors affecting in volume reduction rate

Factors		Mean Volume Reduction Rate (%)	*p*-value
Primary disease	Yes	75.0	0.077
AML	No	56.9	

RT site	Yes	75.7	0.254
Soft tissue	No	64.1	

Concurrent	Yes	78.7	0.248
Chemotherapy	No	65.3	

Initial size	Yes	48.4	0.065
BP ≥ 30 cm^2^	No	72.5	

Initial size	Yes	58.5	0.175
LD ≥ 6.0 cm	No	73.0	

Follow up time	Yes	69.0	0.877
≥ 2 weeks	No	67.3	

Total dose	Yes	76.6	0.178
≥ 25 Gy	No	63.0	

BED_10_	Yes	76.9	0.140
≥ 30 Gy	No	62.2	

*Abbreviations: *AML = Acute myeloid leukemia, BP = Bidimensional product, LD = Largest dimension, BED = Biologic effective dose

### Overall survival

**Figure 2:**
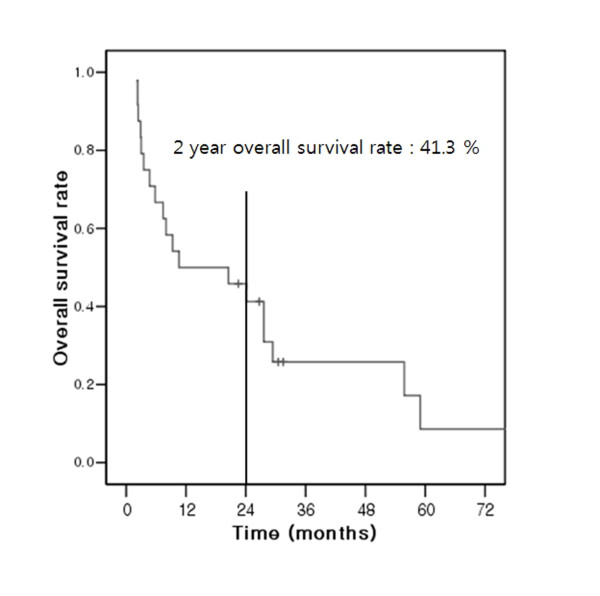
**The overall survival curve**.

The overall survival in patients with EML was poor; only five of 24 patients are still alive. The actuarial median survival time was 11.5 months (range: 2.3-59.0) after the diagnosis of EML. The 1 and 2-year overall survival rate was 50.0% and 41.3%, respectively (Figure 2).

## Discussion

The rare incidence of EML and the variable location of the lesions resulted in a limited clinical experience. Although, EML can occur in any organ or tissue throughout the body, in our study, the most common site was the bone. Soft tissue and lymph nodes were also the major sites of involvement.

The median survival of patients with EML in our study was 11.5 months. It is well known that the presence of EML in leukemic patients is generally associated with a poor clinical outcome with a shorter survival time [1,3,14]. The development of EML may imply that some important signaling pathways exist, which are associated with migration of leukemic cells to the extramedullary organs and tissues.

Therefore, even though the treatment of EML is not well established, many authors suggest that intensive chemotherapeutic agents are the cornerstone in the treatment [4,13,14]. Lan reported that patients undergoing chemotherapy for EML had a significantly longer survival than those not receiving chemotherapy [14]. Yamauchi also suggested that the use of intensive chemotherapy can reduce the risk of subsequent development of leukemia in non-leukemic granulocytic sarcoma patients [4]. On the other hand, the role of RT in the treatment of EML is not well established. Tsimberidou reported RT does not improve the overall or failure-free survival [13]. However, in clinical situations, other treatment modalities except chemotherapy, including surgical decompression or RT, are also necessary on an individual basis for the management of pain and/or other symptoms. Also in some cases, there are patients who cannot undergo chemotherapy because of their poor medical condition [14].

RT is preferred to surgery in many cases because it is non-invasive and leukemic cells are known as extremely radiosensitive. However, the optimal irradiation dose has not been established because of the limited clinical experience. There are some reports treated the EML successfully with RT with or without chemotherapy [4,7,13,14,16-22]. However, the many reports dose not describe the radiation technique or dose. Although, response rates of leukemic infiltrates have been reported with doses as low as 4 Gy, the need for higher doses up to 40 Gy in certain locations is also recognized [23,24]. In a recent report by Bakst et al. [17], they recommend 24 Gy in 12 fractions. In our study, almost all patients received more than 20 Gy except for one patient whose treatment was interrupted due to a poor medical condition. The overall response rate was 76.2% and the symptom response rate was 85.7%, which suggests that in almost all cases the palliation aim can be achieved with RT. Although, there was no correlation with symptom control and RT dose, the overall response rate was better in those treated with more than 25 Gy (or BED_10 _30 Gy) or in those with an initial tumor size smaller than 10 cm^2^.

A CR was achieved in one third (35.8%) of the sites treated, and there is a higher chance of a CR when the tumor is located in the soft tissue. However, when the tumor is large (more than 6 cm or 30 cm^2^), it seems that a higher RT dose is needed. Some authors have also suggested a relationship between the size of the tumors and the total irradiation dose [24].

The limitation of this study was its retrospective study design without controls, small case numbers and insufficient medical record of symptom change. However, this is an early study that focuses on the role of radiation for the treatment of EML in leukemic patients.

## Conclusions

In conclusion, from this retrospective study, we conclude that EML can be managed successfully with RT. The tumor response rate was excellent and symptom relief was achieved in almost all patients, at least in the short term. Although, there was no definite correlation between volume reduction and total dose, it seems that a higher total dose of more than 25 Gy or BED_10 _30 Gy is needed for a better response. We also suggest that a higher total radiation dose may be needed when the EML is large or located in non-soft tissue sites. In addition, we suggest that at least 20 Gy may be enough for symptom palliation in leukemic patients with EML. Further studies are needed for detailed RT dose schedule depending upon the tumor size.

## Competing interests

The authors declare that they have no competing interests.

## Authors' contributions

JHS carried out the study and drafted the manuscript. BOC designed the study and gave final approval for publication. SHS and SMC participated in the design of the study and helped to perform the statistical analyses. JHL and HSJ participated in the analysis and the data interpretation. SHS, JHL participated in the data acquisition and analysis. All authors have read and approved the final manuscript.

## Pre-publication history

The pre-publication history for this paper can be accessed here:

http://www.biomedcentral.com/1471-2407/11/428/prepub
